# Suspected retinopathies in Norwegian optometric practice with emphasis on patients with diabetes: a cross-sectional study

**DOI:** 10.1186/1472-6963-8-38

**Published:** 2008-02-08

**Authors:** Vibeke Sundling, Pål Gulbrandsen, Ragnheiður Bragadottir, Leiv S Bakketeig, Jak Jervell, Jørund Straand

**Affiliations:** 1 Department of Optometry and Visual Science, Buskerud University College, Kongsberg, Norway; 2 Helse Øst Health Services Research Centre, Akershus University Hospital and Faculty of Medicine, University of Oslo, Oslo, Norway; 3 Department of Ophthalmology, Ullevål University Hospital Faculty Division Ullevål University Hospital, University of Oslo, Oslo, Norway; 4 Division of Epidemiology, National Institute of Public Health, Oslo, Norway; 5 Professor Emeritus, Bygdøy alle 25 A, 0262 Oslo, Norway; 6 Department of General Practice and Community Medicine, University of Oslo, Oslo, Norway

## Abstract

**Background:**

The scope of optometry differs worldwide. In Norway the vast majority of optometrists perform ophthalmoscopy as part of their routine examinations. The aim of this study was to describe the frequency of *suspected *retinopathies in patients seen for routine optometric examination and to determine how optometrists deal with these patients.

**Methods:**

212 optometrists participated in a questionnaire survey and a practice registration during November 2004 – May 2005. In the practice registration, details for 20 consecutive patient encounters were recorded. Data were analysed by chi-square tests and multiple logistic regression.

**Results:**

All optometrist stated that ocular history taking was an integrated part of their routine examination, while general health and diabetes history were routinely addressed by 59% and 42% of the optometrists, respectively. During the practice registration 4,052 patient encounters were recorded. Ophthalmoscopy was performed in 88% of the patients, of which 2% were dilated fundus examinations. Retinopathy was *suspected *in 106 patients, of whom 31 did not report a previous history of ocular or systemic disease. Old age (75+), hypertension and diabetes strongly predicted retinopathy with odds ratio (95% CI) of 6.4 (4.2 to 9.9), 3.8 (2.4 to 6.0) and 2.5 (1.4 to 4.7), respectively. Diabetic retinopathy was seen in 10% of diabetic patients and *suspected *in 0.2% of patients with no established history of diabetes. Retinopathy was not confirmed in 9 out 18 patients with a history of diabetic retinopathy; seven of these had undergone laser treatment. Out of the 106 patients with findings of retinopathy, 28 were referred to an ophthalmologist or a general practitioner (GP), written reports were sent to a GP in 16 cases, ten patients were urged to contact their GP for further follow up, while 52 were considered in need of routine optometric follow up only.

**Conclusion:**

Optometric practice provides a low threshold setting for detecting cases of ocular disease and retinal manifestations of systemic disease in the population. At present diagnosis of retinopathy in Norwegian optometric practice is unreliable. There are potentials for improving the optometrists' routine examination, their patient management patterns and collaboration routines with medical doctors.

## Background

The scope of optometry differs worldwide [1] and, more specifically, in Europe [2] ranging from dispensing of optical aids to the diagnosis and treatment of certain ocular diseases. In various countries, there is disparity in the legal recognition of optometry as a health care profession. Since 1988 Norwegian optometric practice has been regulated by The Health Personnel Act, which is founded on the principles of responsible conduct.

In the Scandinavian population, retinal disorders are the most common reason for visual impairment (66%), and in the working age population, diabetes represents a leading cause (13%) [3]. The reported prevalence of diabetic retinopathy differs widely [4]. Most people with diabetes will develop some degree of retinopathy, and 11–30% will develop sight threatening retinopathy during the course of their illness [5-9].

Studies have shown optometrists are able to detect and grade diabetic retinopathy[10] and specially trained optometrist perform well when screening for diabetic retinopathy using dilated, indirect ophthalmoscopy [11-13]. The vast majority of Norwegian optometrists perform ophthalmoscopy as part of their routine examinations [14], and dilated fundus examination can be undertaken by optometrists certified to use ocular diagnostic drugs. Norwegian optometrists with specific certification were given the privilege to acquire and use ocular diagnostic drugs in 2004. At the time of the study 9% of Norwegian optometrists had this privilege, which requires approved education in the use of ocular diagnostic drugs.

There are few studies describing diagnosis and management of retinopathy in routine optometric practice. The aim of this study was to establish the prevalence of possible retinopathy in diabetic and non-diabetic individuals seen in routine optometric practice, to determine the proportion of previously unknown ocular and systemic disease and, finally, to explore how optometrists deal with such patients during everyday practice. The study did not assess or validate the optometrists' findings.

## Methods

**Figure 1:**
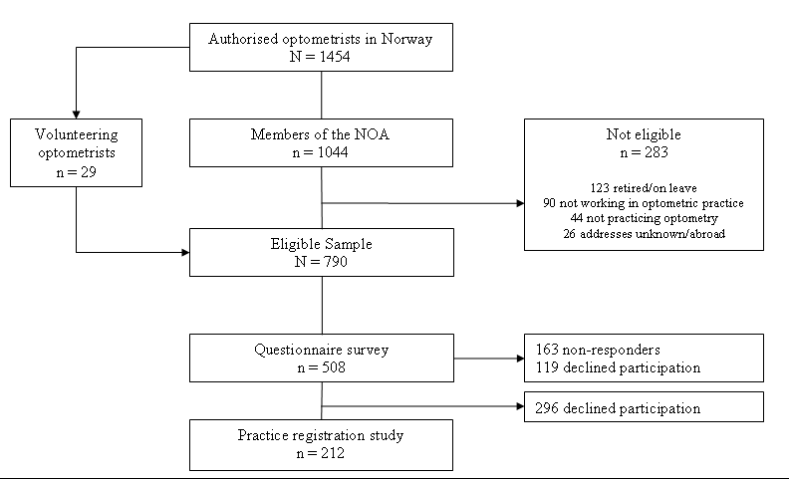
Selection of optometrists in the study.

All members of the Norwegian Association of Optometrists (NAO) working in optometric practice in the community were invited to participate in a questionnaire survey. In addition, 29 practicing non-member optometrists who heard about the study volunteered to participate, making the total sample 790, figure 1. All questionnaire responders (n = 508) were also asked to take part in a practice registration. During November 2004 – May 2005, 212 Norwegian optometrists participated in both the questionnaire survey and the practice registration. The survey has been described elsewhere [14].

In the questionnaire, the optometrists were asked about their education and work experience, practice habits (history taking and examination), opinions on important principles of practice and their collaboration with general practitioners (GPs) and ophthalmologists. In the practice registration, each optometrist recorded the following data for 20 consecutive patients seen for a full eye examination: demographics, patient's history, best corrected visual acuity, intra-ocular pressure, ocular diseases, and how the patients were dealt with (e.g. referral, written report to physicians). Data were reported by the optometrists on a registration form. Recorded ocular diseases were: patient-reported history of cataract, glaucoma and/or age related macular degeneration (AMD) and *suspected *cataract and/or suspected retinopathy. In Norway diagnosis of ocular disease is not in the scope of optometric practice and the terms *suspected *or *possible *retinopathy are used to reflect that these are tentative diagnosis as reported by the optometrists. Additionally patient reported history of: hypertension, cardiovascular disease and diabetes were recorded. In patients with history of diabetes were also asked about type of diabetes, illness duration, treatment, HbA1c-values, blood pressure, diabetic retinopathy, and laser treatment.

The study was presented to Regional Committee for Medical Research Ethics; the study was not regarded subject to specific evaluation and approval. The Norwegian Social Science Data Services were notified prior to commencement of the study. A notice was posted in the consulting room/practice notifying patients of the ongoing practice registration. Patient data was unidentified before it was passed on to the research team and the responding optometrists were anonymous to the researchers.

Differences between proportions were analysed using chi-square tests. Features associated with suspected retinopathy were analysed by univariate and multiple logistic regression. The statistical package SPSS version 12.0.2 was used.

## Results

All optometrists reported that a history of vision and ocular health was part of their routine examination. Respectively, 59% and 42% of the optometrists also addressed general health and diabetes in the patient history taking for *all *patients. Ophthalmoscopy was part of the routine examination for the majority of optometrists (96%). One out of four optometrists was qualified to perform dilated fundus examination. Direct ophthalmoscopy was most frequently used (60%). One out of four reported slit lamp indirect ophthalmoscopy as the most frequent method and one out of ten used *both *direct and indirect ophthalmoscopy in most patients.

**Figure 2:**
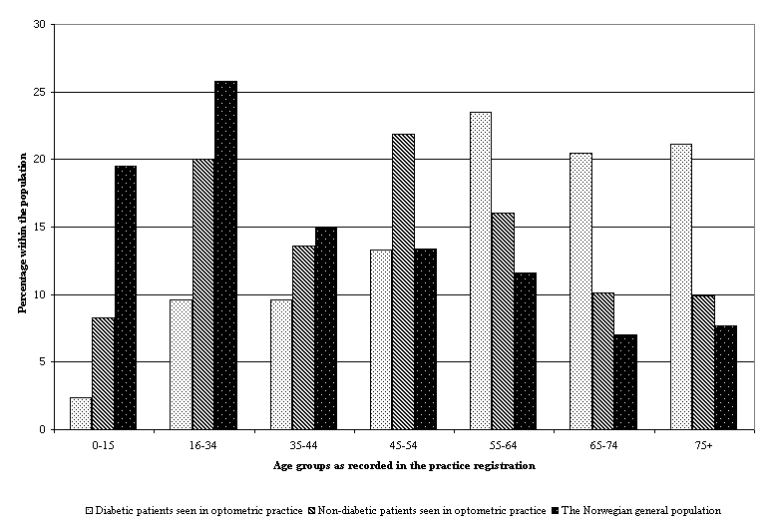
Age distribution of diabetic and non-diabetic patients seen in Norwegian optometric practice compared to the age distribution of the Norwegian population.  Diabetic patients seen in optometric practice,  Non-diabetic patients seen in optometric practice,  The Norwegian population.

During the practice registration, 4,052 patient encounters were recorded, 2,216 (57%) with females. The patients' age distribution is shown in figure 2. Among the patients, 166 had a known history of diabetes, 439 had known hypertension, while 125 had some other known cardio-vascular disease (hypertension excluded). In patients with a history of diabetes, 34 reported a known history of hypertension and 14 reported a known history of other cardio-vascular disease (hypertension excluded).

Ophthalmoscopy was performed in 3,576 (88%) of the patients, of which 78 (2%) were dilated fundus examinations. In patients with known diabetes, ophthalmoscopy was performed significantly more often than in non-diabetics (96% vs 88%, p = 0.002). Tentative retinopathy was found in 106 (3%) patients, of whom 57 (59%) were females. Almost half of these patients were 75 years or older, and none were younger than 16 years. In patients with diabetes, 35% of the *possible *retinopathies were found in the age group 16–64 years. There were no statistically significant differences with regard to gender, age, and known history of hypertension and/or cardiovascular disease between diabetic and non-diabetic patients with findings of retinopathy.

The most common tentative diagnosis made during fundus examination was macular disease (Table 1). More than half of the patients had no previous history of AMD. Diabetic retinopathy was *suspected *in 23 patients, among whom six had no established history of diabetes and 14 had no previous history of retinopathy. In patients with *suspected *hypertensive/vascular retinopathy, 10 out of 27 had no history of hypertension and/or cardiovascular disease and none had a history of diabetes.

**Table 1:** Clinical findings in 3,576 fundus examined encounters and management* by tentative diagnosis and history.

**Optometrists' tentative diagnosis and patients' history**	**n**	**Referral/report/patient urged to contact doctor**	**No/routine optometric follow up**
**Diabetic retinopathy**	**23**	**12**	**11**
No history of retinopathy	14	8	6
History of retinopathy	9	4	5

**Hypertensive/vascular retinopathy**	**27**	**16**	**11**
No history of retinopathy	26	15	11
History of retinopathy	1	1	

**Macular disease^†^**	**56**	**26**	**30**
No history of retinopathy	31	18	13
History of retinopathy	25	8	17

**All retinopathies**	**106**	**54**	**52**

**No retinopathy**	**3,470**	**385**	**3,085**

* Fisher's Exact Test p < 0.001 between patients with findings of retinopathy and patients with no findings of retinopathy

^† ^Fisher's Exact Test p = 0.003 between patients with retinopathy findings and known history of retinopathy and patients with retinopathy findings and no history of retinopathy.

Multiple logistic regression analysis showed that old age (75+), hypertension and diabetes were independent predictors of retinopathy (all kinds), with odds ratio (95% CI) of 6.4 (4.2 to 9.9), 3.8 (2.4 to 6.0) and 2.5 (1.4 to 4.7), respectively. For vascular retinopathy only diabetes and hypertension were independent predictors with odds ratio (95% CI) of 7.2 (3.7 to 14.1) and 4.9 (2.6 to 9.3), respectively.

Diabetic retinopathy was seen in 17 (10%) of the diabetic patients, of these nine had reported history of diabetic retinopathy. However, retinopathy was not described by the optometrists in 9 out of 18 patients with reported history of diabetic retinopathy. Seven of these nine patients reported to have undergone laser treatment. There were no significant differences with regard to gender, age, type of diabetes, diabetes treatment, history of hypertension or cardiovascular disease between diabetic patients with findings of retinopathy (n = 17) and diabetic patients with no retinopathy (n = 147).

In total, 439 of the 3,576 (12%) fundus examined patients were judged by the optometrists to be in need of some medical follow up (referral, report or patient consultation) of the ocular findings (Table 1).

Retinopathy was *suspected *in 3% of the patients seen in optometric practice; of whom two thirds had no previous history of retinopathy. More than half of the *suspected *retinopathies were considered to be in need of some further management by a medical practitioner. Patients with retinopathy were more frequently prompted to contact a physician if the retinopathy was previously unknown (41/71 vs. 13/35, p = 0.003). The reason for non-referral of patients with findings of retinopathies was not explored.

## Discussion

In our study population, the proportion of vascular retinopathy seen in non-diabetics was lower than expected according to figures reported in epidemiological studies [15]. This could be due to the low frequency of dilated fundus examinations in our study. Dilated indirect ophthalmoscopy and photographic grading have a higher sensitivity than direct ophthalmoscopy in detection of retinal abnormalities[16,17]. The low frequency of dilated fundus examinations in our study can be explained by the small number of optometrist qualified to perform dilated ophthalmoscopy and the recent introduction of the privilege to acquire and use ocular diagnostic drugs.

Reported prevalence of diabetic retinopathy varies widely. In Scandinavia, prevalence between 13.8 and 75.1% have been reported in people with diabetes [4], this is higher than the proportion detected by Norwegian optometrists in their practice. However, we do not know how well diabetic patients seen in optometric practice correspond with the diabetic population in the community. The lower number of retinopathies among diabetics seen in optometric practice may reflect a selection bias; diabetic patients should have their retinas regularly examined by an ophthalmologist according to guidelines [18]. Diabetic patients with retinopathies may therefore be less likely to go to an optometrist.

Nine reported cases of retinopathy were not described by the optometrists; however, most of these patients had undergone laser treatment. A possible explanation may be that scarring from laser treatment has not been regarded as retinopathy by the optometrists. However, the retinopathies not detected by the optometrists and the overall low numbers of retinopathies observed among both non-diabetics and diabetics may also represent a poor diagnostic sensitivity. Unfortunately, our data did not permit us to validate the quality of the optometrists' diagnostic work.

The optometrists' follow-up decisions in patients with findings of retinopathy should raise some concern. Only one quarter of the patients with *suspected *vascular retinopathy and no known history of retinopathy or related systemic disease were only considered to be in need of optometric routine follow up. This practice is probably not acceptable. In general, these patients should be seen by a physician as retinal microvascular changes are related to long-term hypertension, type 2 diabetes, impaired glucose metabolism, obesity, dyslipidemia, stroke and an increased cardiovascular mortality [19]. This may suggest that optometrists make *medical *judgements and that patient management depends on their evaluation of the ocular findings, not solely on the patient's history. However, our numbers are low and the reason for non-referral has not been recorded in the study, the interpretation should therefore be considered with caution. If some optometrists do take inappropriate medical responsibility, one possible explanation could be inadequate report and referral routines and lack of established collaboration with medical practitioners.

Previous studies of optometrist's effectiveness in screening for diabetic retinopathy have revealed a specificity ranging from 62 to 95% and a sensitivity of 70 to 87% [11-13,17] Based on the reported prevalence of diabetic retinopathy in the Norwegian diabetic population (13.8%) [20] and the number of retinopathies missed (n = 9) and detected (n = 17) by the optometrists in this study, we propose that the diagnostic specificity must be high. It is unlikely that report/referral of cases of *suspected *retinopathy will impose undue pressure on the health care services. This is supported by a previous study by Riise et al [21] which concluded that 94% of referrals form Norwegian optometrists were clinically relevant. However, taking the low diagnostic sensitivity into consideration suggests that the routine examination as currently undertaken by Norwegian optometrists is an unreliable method of screening for diabetic retinopathy. Moreover, the study illustrates the disparity of optometric practice in Europe and worldwide with regard to training and the role in the health care system, emphasizing the importance that health policies decisions are founded on the practice in the community were the policy will be employed.

Some limitations of the study should be taken into consideration. First, as compared to the non-participants, the optometrists who took part in this study tended to be younger, more were females, and they had in general higher education and worked in smaller communities[14]. Hence their frequency of retinal examinations, method of ophthalmoscopy and collaboration habits may differ from that of the non-participants. Second, practice registration data was recorded for consecutive patients to avoid selection bias, however, the reported patient histories relied on patients' self-report and memory recall. Third, the practice registration may have influenced the way the optometrists performed their routine examination. Finally, we did not observe the optometrists' work and their conclusions were not verified.

## Conclusion

Optometric practice is a low threshold setting for case-finding of ocular pathology and retinal manifestations of systemic disease in the population. At present, the diagnosis of retinopathies in Norwegian optometric practice is unreliable. There are potentials for improving the optometrists' routine examination, their patient management patterns and collaboration routines with medical doctors.

## List of abbreviations used

AMD: Age related macular degeneration; CVD: Cardio-vascular disease; GP: General practitioner; HTN: Hypertension; NAO: Norwegian Association of Optometrists.

## Competing interests

The authors declare that they have no financial competing interests.

VS is a member of the Norwegian Optometric Associations' board of continuing education and board of optometric rehabilitation.

## Authors' contributions

VS conceived of the study and participated in its design, acquisitioned and statistically analysed the data and drafted the manuscript. PG participated in the design of the study and interpretation of data, and helped to draft the manuscript. JS participated in the design of the study and helped to draft the manuscript. RB, LSB and JJ participated in the design of the study and critically revised the manuscript. All authors read and approved the final manuscript.

## Pre-publication history

The pre-publication history for this paper can be accessed here:


